# Supplementary material for: Epidemiology and comorbidity of juvenile idiopathic arthritis in Poland– a nationwide study
Source: Pediatr Rheumatol Online J. 2025 Mar 28;23:33. doi: 10.1186/s12969-025-01065-8 (PMC11951515; doi:10.1186/s12969-025-01065-8)
Supplement: Supplementary file 1 — Supplementary Material 1 [file 12969_2025_1065_MOESM1_ESM.docx]

**Epidemiology and comorbidity of juvenile idiopathic arthritis in Poland – a nationwide study**

Zbigniew Żuber1*, Krzysztof Podwójcic2, Mateusz Szeląg2, Magdalena Krajewska-Włodarczyk3, Krzysztof Batko4, Michał Orleański2, Jakub Sowiński2, Maria Świderek2, Agata Śmiglewska2, Michał Maluchnik5, Marek Brzosko6, Brygida Kwiatkowska7, Marcin Stajszczyk8, Bogdan Batko9,

^1^Department of Pediatrics, Faculty of Medicine and Health Sciences, Andrzej Frycz Modrzewski University, Krakow, Poland [zbyszekzuber@interia.pl](mailto:zbyszekzuber@interia.pl) (Z.Z.)

^2^Department of Analysis and Strategy, Ministry of Health, Poland, [mszelag@mz.gov.pl](mailto:mszelag@mz.gov.pl) (M.S, M.O., K.P., J.S., M.Ś) [m.orleanski@mz.gov.pl](mailto:m.orleanski@mz.gov.pl), [k.podwojcic@mz.gov.pl](mailto:k.podwojcic@mz.gov.pl), [j.sowinski@mz.gov.pl](mailto:j.sowinski@mz.gov.pl), [m.swiderek@mz.gov.pl](mailto:m.swiderek@mz.gov.pl)

^3^Clinic of Rheumatology, School of Medicine, Collegium Medicum, University of Warmia and Mazury, Olsztyn, Poland [magdalenakw@op.pl](mailto:magdalenakw@op.pl) (M.K-W.)

^4^Department of Dermatology, University Hospital, Krakow, Poland [batko.krzysztof@gmail.com](mailto:zbyszekzuber@interia.pl) (K.B.)

^5^Department of Adult Neurology, Medical University of Gdansk, Gdansk, Poland ([michal.maluchnik@gmail.com](mailto:michal.maluchnik@gmail.com), Michał Maluchnik)

^6^Department of Rheumatology, Internal Diseases, Geriatrics and Clinical Immunology, Faculty of Medicine and Dentistry, Pomeranian Medical University, Szczecin, Poland [marek.brzosko@pum.edu.pl](mailto:marek.brzosko@pum.edu.pl) (M.B.)

^7^Clinic of Early Arthritis, National Institute of Geriatrics, Rheumatology and Rehabilitation, Warsaw, Poland. [dyrektor.kliniczny@spartanska.pl](mailto:dyrektor.kliniczny@spartanska.pl) (B.K.)

8Department of Rheumatology and Autoimmune Diseases, Silesian Center for Rheumatology, Orthopedics and Rehabilitation, Ustroń, Poland

^9^Department of Rheumatology and Immunology, Faculty of Medicine and Health Sciences, Andrzej Frycz Modrzewski University, Krakow, Poland [bpbatko@gmail.com](mailto:bpbatko@gmail.com)

Corresponding author: Zbigniew Żuber, Department of Pediatrics, Faculty of Medicine and Health Sciences, Andrzej Frycz Modrzewski Kraków University, 1 Herlinga-Grudzińskiego St., 30-705 Kraków, Poland. e-mail: [zbyszekzuber@interia.pl](mailto:zbyszekzuber@interia.pl)

Abstract

Background: Diagnostic pathways for patients with juvenile idiopathic arthritis (JIA) have gradually improved over time. Provider practice has also shifted towards goal-oriented treatment with disease-modifying drugs (DMARDs) that together may have changed the epidemiologic landscape of JIA.

Methods: Public healthcare utilization records from the National Health Fund (NHF) were screened between 2010-2022. For individuals aged <16 years, we utilized a narrow JIA case definition combining repeat ICD-10 encoding with DMARDs prescription based on ATC codes.

Results: In 2022, we identified 1,625 incident and 29,758 prevalent JIA cases (<16 years), which corresponds to incidence (IRs) and prevalence rates of 4.30 and 78.80 per 100,000 persons of the general population. For the pediatric population, annual IRs for JIA (<16 years) ranged between 24.0 (95% CI 22.8, 25.2) and 38.7 (95% CI 37.2-40.3) per 100,000. Greater susceptibility among females was also consistently observed with the annual IR ratio ranging between 1.16-1.53. The most common concurrent disorders based on medical care services were allergic rhinitis (N=5,200, 17.5%), bronchial asthma (N=3,661, 12.3%) and chronic tonsillitis/pharyngitis (N=3641, 12.2%). Analysis of 214,285 outpatient care visits revealed a median (IQR, range) annual healthcare cost of 37.8€ (35.8-47.4€, 30.3-86.1€) per JIA patient.

Conclusions: This comprehensive, nationwide study provides a contemporary estimate of JIA burden in Poland. Our findings indicate that both the occurrence of new cases and overall burden of JIA in the past ten years align with the lower end of projected figures for our geographical area, especially when compared with Scandinavian nations.

Keywords: disease occurrence; epidemiology; incidence; inflammatory arthritis; juvenile idiopathic arthritis; morbidity; prevalence.

Key messages

- In comparison to higher welfare nations, comprehensive data on JIA epidemiology from lower income countries in Europe are scarce
- This nationwide, healthcare system analysis examines temporal changes in JIA burden of the Polish population, including co-morbidities and direct costs of ambulatory care
- Cyclic and age-dependent trends in incidence rates were observed, with significant comorbidity recorded for every fourth patient with JIA

1. Introduction

In pediatric care, juvenile idiopathic arthritis (JIA) represents the most common type of inflammatory arthritis[1]. On a nosological level, JIA is a clinical concept that encompasses several complex and heterogeneous conditions characterized with arthritis persisting for six weeks or more, which present in patients under the age of 16. The most widespread criteria of the International League of Associations for Rheumatology (ILAR) allow for categorization into seven distinct subtypes based on joint count, serology, and associations with features like uveitis, psoriasis or enthesitis. However, over time, patients transition to different phenotypes, manifesting new symptomatology, while others remain undifferentiated[2].

The global incidence of JIA is estimated between 1 - 23 cases per 100,000 pediatric subjects[3,4]. While no specific geographic predominance is observed, the majority of studied populations originate from Europe and North America[2], with a comprehensive review reporting a pooled incidence of 8 per 100,000 Caucasian children[5]. There are no population-based studies in Poland that quantify the epidemiologic burden of JIA. Prior retrospective estimates reported for 2008-2010 relied solely upon singular ICD-10 encoding sourced from the National Health Fund (NHF) claims[6].

Autoimmune diseases are more prevalent in patients with JIA (across all age groups), while not many large studies exist that have provided a detailed overview of concurrent disorders[7,8]. Previous studies have reported varying rates of comorbid diseases in JIA patients. One Italian study found that 15% of JIA patients had at least one additional autoimmune disease, most commonly thyroid disease[9]. The co-existence of multiple autoimmune diseases in JIA patients may be explained by common genetic factors, as can be inferred from higher prevalence among relatives of JIA patients[10]. Notably, strong associations have been found between JIA and variants in genetic profiles, which are also associated with other autoimmune diseases, such as rheumatoid arthritis[11]. This carries implications for transitional patterns, which remain a poorly studied topic in pediatric rheumatology[12,13].

The transition to stringent control and goal-directed therapy has changed management of rheumatic disorders under the treat-to-target (T2T) approach. In contrast to adult forms of arthritis, the evidence in JIA accumulates with certain lag[14,15], with international recommendations by Ravelli et al.[16] for T2T utilization published only in 2018. Despite advances in treatment, longitudinal cohort studies still report suboptimal outcomes in JIA, with a high burden of disease in adulthood[17].

Management of JIA in Poland is provided by pediatric rheumatologists, but the division to pediatric and adult specialists complicates care for transitional age patients. Transitional JIA management is a difficult problem that is recognized globally[18,19]. Rheumatologists often exhibit variable practice patterns, not always compliant with recommendations, which stems (at least in part) from both medical and non-medical barriers[20]. Details on rheumatology in Poland, including treatment available and healthcare organization are available elsewhere[21].

This nationwide study aims to provide comprehensive epidemiological evidence on the population burden of JIA in Poland. By analyzing public healthcare data from 2010-2022, this report seeks to quantify incidence and prevalence rates, examine age-specific patterns, and identify common concurrent disorders associated with JIA. Additionally, this study also aims to evaluate healthcare system costs for outpatient JIA care.

**2. Methods**

This is a nationwide, retrospective study based on electronic reimbursement claims. The recruitment pool considered all records of healthcare contact in either ambulatory or inpatient services offered by hospitals, clinics and health centers that hold a contract with the National Health Fund and were available for analysis. Inclusion of more recent data was not feasible due to a lag time between service registration, collection and aggregation of data from multiple centers on a national scope. The Polish healthcare system is centralized and based on the Semashko model, with mandatory health insurance that is state supported[22]. A more detailed description of the healthcare system, including diagnostic pathways for JIA, are detailed in Supplementary Data S1.

Definitions

To balance bias of the diagnostic process, but also maintain sensitivity, we developed an expert-based, operating case definition for JIA after consultation with several practicing pediatric rheumatologists, but also general practitioners. The surrogate measure for JIA diagnosis was based on repeat diagnostic records using the ICD-10 classification for inflammatory arthritis encoded as a primary cause for services rendered within public healthcare. All visits required an ICD-10 code compatible with the suspicion of inflammatory arthritis (Table 1) and recorded according to the following criteria:

1. at least 2 visits in outpatient rheumatology care, with a minimum interval of 90 days between visits,
2. at least 1 visit with a general practitioner (GP) or specialist rheumatologist and realization of at least 1 prescription for any of the following arthritis-specific medications: cyclosporine, methotrexate, leflunomide, sulfasalazine, chloroquine or hydroxychloroquine,
3. at least 1 visit in inpatient care,

The patients were classified as JIA at the time of case definition fulfillment; the visit at which at least one of the enrollment criteria (i-iii) was met, which also determined the proxy diagnosis date (ie, if two visits were required, the date when the second visit occurred was treated as the index event). If a patient satisfied multiple definitions, the earliest occurring event determined the disease onset date. All patients were required to fulfill the case definition prior to 16 years of age, to limit the overlap with rheumatoid arthritis or psoriatic arthritis. We also tested an alternative definition with maximum age of 17 years at diagnosis. However, due to very similar trends and possibility of greater bias (16 to 18 years represents transitional age), we retained the more conservative definition (< 16 years of age).

**Data Collection and Preparation**

Utilizing the adopted case definition, we determined the absolute number of incident and prevalent JIA cases between January 1, 2010 – December 30, 2022 from a yearly perspective, as well as any events of death recorded among JIA patients. All information was sourced from the repositories of the National Health Fund and integrated databases by specialist analytics from the Ministry of Health.

To better understand changes within the population burden, we calculated epidemiological measures: annual period prevalence (PR) based on the total number of JIA cases collected up to December 31 of each year, respective to the population at risk (general population; GP) and multiplied by 100,000. Similarly, annual incidence rates (IR) were calculated by dividing the number of new cases observed each year. Once recorded, each JIA case was counted with the prevalent pool, while the total cohort was limited to living patients at the latest available date.

Analysis and visualization were performed using R version 4.4.1 (R Core Team, 2024, R Foundation for Statistical Computing, Vienna, Austria) with the use of publically available tidyverse for data manipulation and cleaning. Poisson-based 95% CI intervals were calculated for age-specific PRs and IRs using the epitools package.

Due to the distinct structuring of healthcare into adult and pediatric, costs were calculated for patients fulfilling the JIA definition up to 18 years of age. Owing to lump sum reimbursement (ie, fixed pricing) and regulatory changes in pricing for services in general practice and inpatient care, cost calculations were not possible to reliably determine in total. The presented cost calculations refer to all forms of ambulatory care provided for JIA patients by specialists. A tabular summary with annual breakdown of costs on a patient and ambulatory care visit level is provided in Supplementary Data 2.

**Ethical approval**

This analysis relies solely on data obtained from the National Health Fund electronic databases. Obtaining institutional ethical approval was not required. Data utilized for this analysis was fully anonymized, which precludes the ability to reliably identify individuals.

**3. Results**

**3.1 Temporal Trends in Population Burden of Juvenile Idiopathic Arthritis**

Using the definition of JIA <16 years, in 2022, we identified a total 29 758 individuals with JIA, with 1,625 newly detected cases. This corresponds to an IR and PR of 4.30 and 78.80 per 100,000 persons of the GP, respectively. The IR for the pediatric population <16 years was estimated at 25.8 (95% CI 24.6-27.1). The most common ICD-10 claims that were recorded to fulfill this case definition for JIA were: M08.9 (22.3%), M08.8 (20.6%), M08.0 (15.5%), M08.4 (13.6%), M08 (11.7%), M08.3 (4.7%), M08.2 (2.4%), M06 (1.7%), M06.9 (1.2%) and M06.4 (1.2%). For a detailed overview of annual IRs and PRs (<16 years) over time, see Table 2.

We further examined temporal changes in PRs of JIA (ever diagnosed, <16 years) by age. In general, the prevalence of JIA appears to be consistently increasing in Poland (Figure 1). However, if we examine the incidence of JIA within the pediatric population (<16 years), a decremental trend can be observed, though the decline coincides with the years of the COVID19 pandemic (Figure 2). To better understand epidemiologic dynamics, we examined IRs and PRs across pooled age groups (Figure 3). Between 2010-2022, incremental trends were observed for adolescents (13-18 years), while conversely, a plateau phase was apparent for younger (2-6 years), as well as older children (7-12 years). In contrast, IRs were similar across age groups (excluding extreme groups – ie, infants and adults), with a moderate declining trend.

Overall, annual IRs for JIA (<16 years) ranged between 24.0 (95% CI 22.8, 25.2) and 38.7 (95% CI 37.2-40.3) per 100,000 pediatric patients below 16 years old (Figure 2). Greater susceptibility among females was also consistently observed (Figure 4), with the annual IRR ranging between 1.16-1.53.

In additional analyses using an extended definition of JIA (<18 years), the corresponding number of incident and prevalent JIA patients was estimated at 1 812 and 34 180, with IR and PR of 4.80 and 90.50 per 100,000 persons of the GP, respectively. If we evaluate potential trends in IRs and PRs for age groups approaching adulthood (Figure 3), an increase in the total number of JIA cases can be observed.

At the latest timepoint available (2022), we observed that following transition to adult care (>18 years), most JIA patients were not recorded with diagnoses of adult inflammatory arthritis (N=10 289, 66.9%). Out of the remaining cases, the most frequent transitional patterns were rheumatoid arthritis (N=3813, 24.8%), psoriatic arthritis (N=775, 5.0%), ankylosing spondylitis (N=812, 5.3%), Still’s disease (N=78, 0.5%) and inflammatory axial disease (N=430, 2.8%). In some cases, multiple different coding structures were observed in a given patient, which may reflect an ongoing diagnostic process.

**3.3 The Most Common Concurrent Disorders in Juvenile Idiopathic Arthritis**

For the JIA population (<16 years), we examined trends in healthcare utilization data to identify patterns of co-morbidity. In a broad perspective (level I ICD-10 class), medical care was most frequently required due to pulmonary (N=12 093, 40.6%), ocular (N=10 811, 36.3%), skin (N=9210, 31.0%), genitourinary (N=5489, 18.4%), genetic/growth abnormalities (N=4957, 16.7%), neurologic (N=4675, 15.7%), infectious (M=4605, 15.5%), ear disorders (N=4568, 15.4%), gastroenterological (N=3913, 13.5%), neoplasms (N=3892, 13.1%), psychiatric (N=2893, 9.7%), cardiovascular (N=2228, 7.5%) and hematologic (N=1801, 6.1%) claims.

Furthermore, we examined a set of 50 level III codes to determine the more specific disorders that are treated among JIA subjects (<16 years). After discarding non-specific codes, but also musculoskeletal disease specific (ie, trauma-related), we observed that allergic rhinitis (N=5200, 17.5%), scoliosis (N=3844, 12.9%), bronchial asthma (N=3661, 12.3%), chronic tonsillitis/pharyngitis (N=3641, 12.2%), acne (N=2879, 9.7%), growth restriction (N=2311, 7.8%), congenital hip disorder (N=2304, 7.7%), viral warts (N=2268, 7.6%), non-purulent middle ear infection (N=2120, 7.1%), atopic dermatitis (N=2014, 6.8%), speech disorder (N=1833, 6.2%), headache syndromes (N=1703, 5.7%), heart murmur (N=1651, 5.6%), reactive arthropathy (N=1646, 5.5%), chronic rhinosinusitis (N=1584, 5.3%), uveitis (N=992, 3.3%), other connective tissue diseases (N=1283, 4.3%) and hypothyroidism (N=1065, 3.6%) were the most frequent concurrent conditions related to JIA. Of note, out of ocular conditions, refractive disorders (21.9%), strabismus (3.5%) and anterior uveitis (3.3%) were most often coded.

To ascertain the severity of comorbidity, we also calculated composite scores that assist in differentiating poor prognosis. When considering the Charlson score, most JIA patients (<16 years) had a low burden of excess morbidity (0 – 22 975, 77.2%; 1 – 6 237, 20.96%; 2 – 517, 1.74%; 3 or more – 29, 0.1%). Similarly, estimates based on the Elixhauser score suggest that only a proportion of JIA subjects suffer from multimorbidity (0 – 8 196, 27.5%; 1 – 16 276, 54.7%; 2 – 4 135, 13.9%; 3 - 906, 3.0%, 4 or more – 233, 0.8%).

**3.4 Healthcare System Costs in Ambulatory Juvenile Idiopathic Arthritis Care**

After extracting data from 214 285 visits, we calculated systemic costs of JIA treatment (<18 years; wider definition due to organizational construct) based on claims from outpatient visits (see Supplementary Table S1 and S2). The annual median (IQR, range) cost was estimated at 37.8€ (35.8-47.4€, 30.3-86.1€, respectively) for one patient with JIA treated in outpatient care. For the Polish healthcare system, between 2010-2022, the total cost of outpatient JIA treatment amounted to over 3 540 000€, with a median (IQR, range) cost of 232 000€ (178 000-309 000€, 103 000-573 000€, respectively) per year reimbursed for outpatient service. The median (IQR, range) cost per outpatient visit for a JIA patient was estimated at 14.1€ (12.1-18.3€, 9.70-33.8€), respectively.

**4. Discussion**

This is the first robust study that quantifies the epidemiological burden of JIA in Poland between 2010-2022. We utilized a complex case definition, which combines repeat ICD-10 encoding, prescription data and accounts for hierarchical structure in healthcare. To date, there have been no population-based studies for JIA in Poland. Previously, estimates were derived from an observational, cohort study conducted on a regional level[23]. Another prior domestic report for 2008-2012 aimed to evaluate JIA burden, though the authors focused on regional and rural-urban differences, while also utilizing singular ICD-10 claims to identify JIA[6]. In this study, we observed that incidence of JIA is stable and potentially decremental over time. Whether this corresponds to a true decrease incidence or reflects healthcare restriction in the pandemic years (among other potential confounding factors) is currently unknown. However, with the often-chronic nature of JIA, the prevalent population of patients is growing (on par with the aging general population).

In broad terms, our findings are comparable with those of other studies. Recent studies from the UK reported an overall age-standardized incidence rate at 5.61 and prevalence rate of 43.5 per 100 000 persons in 2018[24]. Using a database of 3-4 mln patients, Horneff et al. reported incidence rates between 34-60 and prevalence rates between 133-168 per 100 000 persons[25]. In Nordic countries, incidence and prevalence rates tend to be higher (18.5 and 159, respectively). A prior systematic review synthesized the pooled incidence and prevalence rates with an estimate of 7.8 (95% CI 7.6, 8.1) and 20.5 (95% CI 19.8, 21.3), respectively (of note, data mainly from Europe and North America’s, without distinction by JIA subtype)[5]. In historical cohorts (1990’s and earlier), the prevalence was estimates ranged between 60 – 400 per 100 000 for JIA[26]. A recent study from Finland estimated JIA occurrence at 27.5–31.7 per 100,000 per year, which is higher than prior reports (from Finland) and considerably greater than the estimates for Europe, as well as our study[27]. Although prior observations of south-north disparities[28] were not confirmed, considerable regional and inter-country variability[5] implies the importance of both environmental and genetic factors. Of note, the higher values obtained for age-specific IRs and PRs are likely to reflect the changing demographic structure of the population (smaller denominator) and population coverage by public care. Gathering additional epidemiological data is of high importance due to considerable variability on a geographical and ethnic level, both in disease burden and course[29–31].

While JIA may attenuate throughout young adulthood, the majority of patients exhibit ongoing signs of disease[32], and likely transition into undifferentiated or adult form of arthritis. A study by Glerup et al. based on a cohort study from Scandinavian countries shows the high burden of disease that persists into adulthood, with only one third of patients achieving remission, despite being diagnosed in the early biologic era[17]. Delayed introduction of biologic DMARDS leads to enhanced disability and quality of life, including lower chances for drug-free remission after transitioning into adulthood[33]. In contrast to earlier epidemiologic studies, we did not restrict the scope of the investigation to individuals 16 years or younger, as we know little about transitional age JIA. We observed that a substantial proportion of JIA patients are young adolescents or adults, which is important from a management perspective. Transition into adulthood is tied to a change in the healthcare provider, but also carries psychosocial considerations. Adherence and illness coping is worse in adolescence[34]. Despite initially high effectiveness of biologics, long term retention is suboptimal[35]. A single monocentric study from Poland showed that adult JIA is highly heterogenous and difficult to treat, with less than 50% of patients meeting criteria for adult types arthritis[36]. Our findings emphasize the population burden, and further showcase the importance of future studies of the transitional JIA population. Due to cross-sectional nature, we are unable to assess the number of JIA patients that reach long lasting remission.

We also examined the most common chronic causes for healthcare contact among JIA patients. Our results are similar to a recent report by Horneff et al. from Germany, who observed the common concurrent presence of atopic dermatitis, allergic diseases and others[25]. However, we did not observe such a high rate of uveitis, as reported by Horneff et al (11%)[25]. While there is not dedicated screening programme in Poland, and there are few potential confounding sources (underreporting, miscoding, diagnostic lag) given the strong association with JIA, this finding may be suggestive of an unmet need that requires urgent clarification in population-based studies[37,38]. Haslak et al. reported allergic rhinitis, attention disorders and atopic dermatitis as the most common concurrent conditions[39]. Other reports from smaller cohorts indicate the common prevalence of uveitis (18%), allergic rhinitis (14.5%), migraine (8.7%) and atopic dermatitis (8.7%)[40], which may markedly differ from population estimates due to sampling heterogeneity. Of note, we did not observe a frequent (ie, within the top 50) reporting of concurrent claims for other, related autoimmune disorders[7,8], which are well recognized to coincide with JIA. Whether resultant from potential latency, underdiagnosis or miscoding, this observation warrants further investigation in the Polish setting.

We observed very low rates of mortality in this study, which is consistent with other reports from Europe. It has been shown that JIA carries a comparable risk of death, as compared with the general population. Although this may not be true for untreated or refractory JIA, the general observations are likely reflective of the high level of care that pediatric patients experience[41–43].

In contrast to the adult form of inflammatory arthritis, the cost of JIA is initially driven by medication costs[43,44]. However, the true burden of disease becomes tractable only after some time (ie, disability and sequelae of its chronicity and immunosuppression), with some authors observing over 50% of JIA patient have active disease later in adulthood[43]. Due to geographic disparities in healthcare across regions, it is necessary to interpret economic calculations respective to the local setting. Yucel et al. emphasized that in systems with low remuneration for professional consultations (excluding laboratory tests and drug costs), the remaining share of total costs can be nearly negligible (3%), despite a high rate of anti-TNF therapy use[44]. Therefore, the reported costs of JIA treatment in Poland are likely only a proportion of total real-life costs (eg, medication copayments, travel costs, parents’ work leave costs), as has been shown in a comprehensive review for low- and middle-income countries[45].

The limitations of this study include retrospective character and utilization of secondary sources to establish a tentative, proxy diagnosis. Epidemiologic calculations are based on the use of a denominator of population size at-risk of developing disease at a given time (per year), which does not always equate to person-years at-risk, which is the ideal case scenario for epidemiologic estimates[46]. While we utilized fixed population size at a given time, the chronicity of JIA and young age distribution limits the bias associated with incomplete follow-up (an imprecise denominator). Of note, we observed higher incidence in 2010 compared to subsequent years, which may be attributed to left truncation, whereby some patients were already diagnosed prior to the study period, resulting in an initial overestimation of incidence that stabilized in later years as the cohort matured. We are unable to account for additional sources of bias such as miscoding, incompleteness or errors within the diagnostic and therapeutic process, which would result in false fulfillment of the JIA definition. Additionally, some of the diagnostic codes that we utilize can be indicative of other forms of arthritis, but were maintained to enhance sensitivity, given their relatively low frequency of use. Due to anonymization of the analyzed data, we are unable to perform true validation using a stratified random sampling approach. Data form studies comparing NHF claims with real-life registry data in adult disorders indicates potential variability in both under- or overestimation of certain conditions. However, these analyses relied solely on primary ICD-10 diagnostic encoding[47]. While the use of a complex case definition and near complete population coverage with public healthcare are the strengths of this report, the validity of these estimates requires further replication in population cohorts. Potential solutions to improve the reliability of NHF records include the use of natural language processing techniques that will emerge in importance as we transition to a comprehensive, central medical documentation platform[47,48]. Lastly, geographical differences are likely to be present throughout Europe, but due to the demographic and ethnic structure of Poland, this cohort is likely representative of Central-Eastern Europe.

**5. Conclusion**

Reliable epidemiological estimates of JIA burden are important for the policy maker in healthcare resource allocation and assist identification of areas for intervention priority. This nationwide study provides the first comprehensive epidemiological assessment of JIA in Poland, highlighting its substantial population burden from 2010 to 2022. The findings suggest a stable yet slightly declining incidence trend, while the prevalent population continues to grow, likely due to the chronicity of the disease. Relatively high rates of coexisting disorders and healthcare utilization from a young age underscore the complex needs of JIA patients. As many JIA patients transition into adulthood forms of inflammatory arthritis, the present study emphasizes the importance of research into the unique characteristics and unmet needs of this population.

**Figure Legends.**

Figure 1. JIA prevalence rate (PR) with Poisson-based 95% Confidence Intervals (95% CI) for each specific childhood age group over time.

Notes: Epidemiologic estimates are calculated with the population at risk as the number of individuals in each specific age group.

Figure 2. JIA incidence rate (IR) with Poisson-based 95% Confidence Intervals (95% CI) within the pediatric population over time.

Notes: Epidemiologic estimates are calculated with the population at risk as the number of individuals with JIA <16 years within the childhood population below 16 years of age.

Figure 3. Temporal changes in JIA incidence rate (IR) and prevalence rate (PR) across pooled age groups.

Notes: Epidemiologic estimates are calculated with the population at risk as the number of individuals in each specific age range.

Figure 4. Gender specific changes in the incidence of JIA over time.

Notes: Epidemiologic estimates are calculated with the population at risk as the number of individuals in each specific age range.

**Table 1.** Disorders compatible with the surrogate definition of juvenile idiopathic arthritis based on corresponding ICD-10 claim codes

| Disorder | ICD-10 code for JIA (<16 years of age) | ICD-10 code for transitional pattern (adults) |
| --- | --- | --- |
| Juvenile idiopathic arthritis | M08 and extension (.0, .1, .2, .3, .4, .8., .9) | NA |
| Juvenile psoriatic arthritis | M09 and extension (.0, .1, .2, .8) | NA |
| Psoriatic arthritis | L40 with extension (.5), M07 (.0, .1, .2, .3) | L40 with extension (.5), M07 (.0, .1, .2, .3) |
| Rheumatoid arthritis | M06 and extension (.0, .1, .2, .3, .4, .8, .9) | M05 and extension (.0, .1, .2, .3, .8, .9); M06 and extension (.0, .1, .2, .3, .4, .8, .9) |
| Other forms of joint inflammation | M13 and extension (.0, .1, .8, .9) | NA |
| Ankylosing spondylitis | M45 | M45;  M46.8, M46.9 (inflammatory axial disease) |

**Table 2**. Annual number of new and prevalent juvenile idiopathic arthritis cases and associated general population rates

| Year | Prevalent cases | Incident cases | Mortality cases | IR per 100 000 | PR per 100 000 | MR per 100 000 |
| --- | --- | --- | --- | --- | --- | --- |
| 2010 | 6 117.00 | 2 163.00 | 1 | 5.61 | 15.88 | 0.00 |
| 2011 | 8 088.00 | 1 972.00 | 1 | 5.12 | 20.99 | 0.00 |
| 2012 | 10 275.00 | 2 190.00 | 3 | 5.68 | 26.67 | 0.01 |
| 2013 | 12 380.00 | 2 107.00 | 2 | 5.47 | 32.16 | 0.01 |
| 2014 | 14 766.00 | 2 394.00 | 8 | 6.22 | 38.37 | 0.02 |
| 2015 | 17 044.00 | 2 283.00 | 5 | 5.94 | 44.34 | 0.01 |
| 2016 | 19 263.00 | 2 228.00 | 9 | 5.80 | 50.12 | 0.02 |
| 2017 | 21 341.00 | 2 087.00 | 9 | 5.43 | 55.53 | 0.02 |
| 2018 | 23 294.00 | 1 959.00 | 6 | 5.10 | 60.54 | 0.02 |
| 2019 | 25 216.00 | 1 930.00 | 8 | 5.03 | 65.70 | 0.02 |
| 2020 | 26 583.00 | 1 381.00 | 14 | 3.61 | 69.47 | 0.04 |
| 2021 | 28 147.00 | 1 578.00 | 14 | 4.14 | 73.91 | 0.04 |
| 2022 | 29 758.00 | 1 625.00 | 14 | 4.30 | 78.80 | 0.04 |

Abbreviations: Prevalence Rate, PR; Incidence Rate, IR; Mortality Rate, MR

**Declarations**

Ethics approval and consent to participate: Under local regulations, ethical approval is not required due to the retrospective and secondary nature of the data. Data were also anonymized prior to analysis with subgroup size > 5 cases.

Consent for publication: Not applicable

Availability of data and materials: Raw data is publically available in abridged form on the online platform for Strategic Analyses published by the Ministry of Health, but also available in greater detail upon reasonable request from the authors.

Competing interests: none to declare

Funding: This paper was prepared as a part of the "Maps of Health Needs – Database of Systematic and Implementation Analysis" (grant number POWR.05.02.00-00-0149/15) and ‘Integrated Analytical Platform’ projects (grant number POPC.02.02.00-00-0015/18-00), both co-financed by the European Union from the European Social Fund and European Regional Development Fund respectively.

Author contributions: Conceptualization: ZZ, BB, MK-W, MSz, BK; Methodology: MK-W, MSz, BB, ZZ, KP, BK; Literature review, preparation and writing the original article: MK-W, MSz, MSt, BB, ZZ; Visualisation: MK-W, MSz, KB, KP; Reviewing and editing the manuscript: MK-W, MSz, KB, BB, ZZ, MO, KP, JS, JJ, MŚ, MB, AŚ, BK; Supervision: MK-W, MSz, BB, ZZ, BK. All authors fulfill all authorship criteria by ICMJE and take full responsibility for the accuracy and integrity of all aspects of the work.

Acknowledgements: Not applicable

Abbreviations

- JIA: Juvenile Idiopathic Arthritis
- ILAR: International League of Associations for Rheumatology
- NHF: National Health Fund
- T2T: Treat-to-Target
- GP: General Practitioner
- PR: Prevalence Rate
- IR: Incidence Rate
- ICD-10: International Classification of Diseases, 10th Revision
- COVID-19: Coronavirus Disease 2019
- CI: Confidence Interval
- IQR: Interquartile Range
- UK: United Kingdom
- DMARDS: Disease-Modifying Antirheumatic Drugs
- TNF: Tumor Necrosis Factor

References

1. Petty RE, Southwood TR, Manners P, Baum J, Glass DN, Goldenberg J, et al. International League of Associations for Rheumatology classification of juvenile idiopathic arthritis: second revision, Edmonton, 2001. J Rheumatol. 2004;31:390–2.

2. Palman J, Shoop-Worrall S, Hyrich K, McDonagh JE. Update on the epidemiology, risk factors and disease outcomes of Juvenile idiopathic arthritis. Best Pract Res Clin Rheumatol. 2018;32:206–22.

3. Savolainen E, Kaipiainen-Seppänen O, Kröger L, Luosujärvi R. Total incidence and distribution of inflammatory joint diseases in a defined population: results from the Kuopio 2000 arthritis survey. J Rheumatol. 2003;30:2460–8.

4. Prieur AM, Le Gall E, Karman F, Edan C, Lasserre O, Goujard J. Epidemiologic survey of juvenile chronic arthritis in France. Comparison of data obtained from two different regions. Clin Exp Rheumatol. 1987;5:217–23.

5. Thierry S, Fautrel B, Lemelle I, Guillemin F. Prevalence and incidence of juvenile idiopathic arthritis: a systematic review. Joint bone spine. 2014;81:112–7.

6. Tłustochowicz M, Śliwczyński A, Iltchev P, Brzozowska M, Sierocka A, Marczak M, et al. Juvenile idiopathic arthritis morbidity rate in rural and urban areas of Poland 2008–2012. Ann Agric Environ Med. 2015;22:704–7.

7. Simon TA, Harikrishnan GP, Kawabata H, Singhal S, Brunner HI, Lovell DJ. Prevalence of co-existing autoimmune disease in juvenile idiopathic arthritis: a cross-sectional study. Pediatric rheumatology online journal [Internet]. 2020 [cited 2023 Aug 19];18. Available from: https://pubmed.ncbi.nlm.nih.gov/32503658/

8. Stagi S, Giani T, Simonini G, Falcini F. Thyroid function, autoimmune thyroiditis and coeliac disease in juvenile idiopathic arthritis. Rheumatology (Oxford). 2005;44:517–20.

9. Tronconi E, Miniaci A, Pession A. The autoimmune burden in juvenile idiopathic arthritis. Ital J Pediatr. 2017;43:56.

10. Prahalad S, Shear ES, Thompson SD, Giannini EH, Glass DN. Increased prevalence of familial autoimmunity in simplex and multiplex families with juvenile rheumatoid arthritis. Arthritis Rheum. 2002;46:1851–6.

11. Prahalad S, Hansen S, Whiting A, Guthery SL, Clifford B, McNally B, et al. Variants in TNFAIP3, STAT4, and C12orf30 loci associated with multiple autoimmune diseases are also associated with juvenile idiopathic arthritis. Arthritis Rheum. 2009;60:2124–30.

12. Tan J, Tiller G, Gowdie P, Cox A, Munro J, Allen R, et al. Patterns and rates of confirmed transfer of care of patients with juvenile idiopathic arthritis at a tertiary paediatric rheumatology centre. Intern Med J. 2023;53:46–50.

13. Conti F, Pontikaki I, D’Andrea M, Ravelli A, De Benedetti F. Patients with juvenile idiopathic arthritis become adults: the role of transitional care. Clin Exp Rheumatol. 2018;36:1086–94.

14. Guzman J, Henrey A, Loughin T, Berard RA, Shiff NJ, Jurencak R, et al. Predicting Which Children with Juvenile Idiopathic Arthritis Will Not Attain Early Remission with Conventional Treatment: Results from the ReACCh-Out Cohort. J Rheumatol. 2019;46:628–35.

15. Swart JF, van Dijkhuizen EHP, Wulffraat NM, de Roock S. Clinical Juvenile Arthritis Disease Activity Score proves to be a useful tool in treat-to-target therapy in juvenile idiopathic arthritis. Ann Rheum Dis. 2018;77:336–42.

16. Ravelli A, Consolaro A, Horneff G, Laxer RM, Lovell DJ, Wulffraat NM, et al. Treating juvenile idiopathic arthritis to target: recommendations of an international task force. Ann Rheum Dis. 2018;77:819–28.

17. Glerup M, Rypdal V, Arnstad ED, Ekelund M, Peltoniemi S, Aalto K, et al. Long-Term Outcomes in Juvenile Idiopathic Arthritis: Eighteen Years of Follow-Up in the Population-Based Nordic Juvenile Idiopathic Arthritis Cohort. Arthritis Care Res (Hoboken). 2020;72:507–16.

18. Matsumoto T, Mori M. Questionnaire survey on transitional care for patients with juvenile idiopathic arthritis (JIA) and families. Mod Rheumatol. 2021;31:691–6.

19. Shaw KL, Southwood TR, McDonagh JE, British Society of Paediatric and Adolescent Rheumatology. Young people’s satisfaction of transitional care in adolescent rheumatology in the UK. Child Care Health Dev. 2007;33:368–79.

20. Batko B, Korkosz M, Juś A, Wiland P. Management of rheumatoid arthritis in Poland - where daily practice might not always meet evidence-based guidelines. Arch Med Sci. 2021;17:1286–93.

21. Maślińska M, Dobrzyński T, Kwiatkowska B. Rheumatology in Poland. Rheumatol Int. 2019;39:757–67.

22. Sagan A, Panteli D, Borkowski W, Dmowski M, Domanski F, Czyzewski M, et al. Poland health system review. Health Syst Transit. 2011;13:1–193.

23. Zuber Z, Kania U, Król-Zdechlikiewicz A, Morawska A, Pilch B, Rajska E, et al. Analysis of Clinical Symptoms and Laboratory Profiles in Children with Juvenile Idiopathic Arthritis in Malopolska Region (Poland) in the Years 2007-2010. Open Access Macedonian Journal of Medical Sciences. 2014;2.

24. Costello R, McDonagh J, Hyrich KL, Humphreys JH. Incidence and prevalence of juvenile idiopathic arthritis in the United Kingdom, 2000–2018: results from the Clinical Practice Research Datalink. Rheumatology (Oxford). 2021;61:2548–54.

25. Horneff G, Borchert J, Heinrich R, Kock S, Klaus P, Dally H, et al. Incidence, prevalence, and comorbidities of juvenile idiopathic arthritis in Germany: a retrospective observational cohort health claims database study. Pediatric Rheumatology. 2022;20:1–13.

26. Manners PJ, Diepeveen DA. Prevalence of juvenile chronic arthritis in a population of 12-year-old children in urban Australia. Pediatrics. 1996;98:84–90.

27. Uusitupa E, Rahikkala H, Sard S, Pokka T, Salo H, Kärki J, et al. Incidence of juvenile idiopathic arthritis in Finland, 2000–2020. Rheumatology. 2024;keae322.

28. Berntson L, Andersson Gäre B, Fasth A, Herlin T, Kristinsson J, Lahdenne P, et al. Incidence of juvenile idiopathic arthritis in the Nordic countries. A population based study with special reference to the validity of the ILAR and EULAR criteria. J Rheumatol. 2003;30:2275–82.

29. Kurahara D, Tokuda A, Grandinetti A, Najita J, Ho C, Yamamoto K, et al. Ethnic differences in risk for pediatric rheumatic illness in a culturally diverse population. J Rheumatol. 2002;29:379–83.

30. Kok VC, Horng J-T, Huang J-L, Yeh K-W, Gau J-J, Chang C-W, et al. Population-based cohort study on the risk of malignancy in East Asian children with juvenile idiopathic arthritis. BMC Cancer. 2014;14:634.

31. Saurenmann RK, Rose JB, Tyrrell P, Feldman BM, Laxer RM, Schneider R, et al. Epidemiology of juvenile idiopathic arthritis in a multiethnic cohort: ethnicity as a risk factor. Arthritis Rheum. 2007;56:1974–84.

32. Nordal E, Zak M, Aalto K, Berntson L, Fasth A, Herlin T, et al. Ongoing disease activity and changing categories in a long-term nordic cohort study of juvenile idiopathic arthritis. Arthritis Rheum. 2011;63:2809–18.

33. Oliveira Ramos F, Rodrigues AM, Melo AT, Aguiar F, Brites L, Azevedo S, et al. Influence of the timing of biological treatment initiation on Juvenile Idiopathic Arthritis long-term outcomes. Arthritis Res Ther. 2023;25:177.

34. Kirchner S, Klotsche J, Liedmann I, Niewerth M, Feldman D, Dressler F, et al. Adherence, helpfulness and barriers to treatment in juvenile idiopathic arthritis - data from a German Inception cohort. Pediatr Rheumatol Online J. 2023;21:31.

35. Mourão AF, Santos MJ, Melo Gomes JA, Martins FM, Mendonça SC, Oliveira Ramos F, et al. Effectiveness and long-term retention of anti-tumour necrosis factor treatment in juvenile and adult patients with juvenile idiopathic arthritis: data from Reuma.pt. Rheumatology (Oxford). 2016;55:697–703.

36. Felis-Giemza A, Chmurzyńska K, Kołodziejczyk B, Gazda A. Revising diagnosis of juvenile idiopathic arthritis in adults: a single-center retrospective study. Rheumatol Int. 2023;43:1307–11.

37. Clarke SLN, Sen ES, Ramanan AV. Juvenile idiopathic arthritis-associated uveitis. Pediatric Rheumatology. 2016;14:27.

38. Rypdal V, Glerup M, Songstad NT, Bertelsen G, Christoffersen T, Arnstad ED, et al. Uveitis in Juvenile Idiopathic Arthritis: 18-Year Outcome in the Population-based Nordic Cohort Study. Ophthalmology. 2021;128:598–608.

39. Haşlak F, Guliyeva V, Hotaman B, Duman Ç, Yıldız M, Günalp A, et al. Non-Rheumatic Chronic Comorbidities in Children with Juvenile Idiopathic Arthritis. Turkish archives of pediatrics. 2023;58:212–9.

40. Raab A, Sengler C, Niewerth M, Klotsche J, Horneff G, Zink A, et al. Comorbidity profiles among adult patients with juvenile idiopathic arthritis: results of a biologic register. Clin Exp Rheumatol. 2013;31:796–802.

41. Chhabra A, Robinson C, Houghton K, Cabral DA, Morishita K, Tucker LB, et al. Long-term outcomes and disease course of children with juvenile idiopathic arthritis in the ReACCh-Out cohort: a two-centre experience. Rheumatology (Oxford). 2020;59:3727–30.

42. Kyllönen MS, Kautiainen H, Puolakka K, Vähäsalo P. The mortality rate and causes of death among juvenile idiopathic arthritis patients in Finland. Clin Exp Rheumatol. 2019;37:508–11.

43. Minden K, Niewerth M, Listing J, Biedermann T, Schöntube M, Zink A. Burden and cost of illness in patients with juvenile idiopathic arthritis. Ann Rheum Dis. 2004;63:836–42.

44. Yucel IK, Seyahi E, Kasapcopur O, Arisoy N. Economic impact of juvenile idiopathic arthritis and familial Mediterranean fever. Rheumatol Int. 2012;32:1955–62.

45. de Broucker G, Sim SY, Brenzel L, Gross M, Patenaude B, Constenla DO. Cost of Nine Pediatric Infectious Illnesses in Low- and Middle-Income Countries: A Systematic Review of Cost-of-Illness Studies. Pharmacoeconomics. 2020;38:1071–94.

46. Spronk I, Korevaar JC, Poos R, Davids R, Hilderink H, Schellevis FG, et al. Calculating incidence rates and prevalence proportions: not as simple as it seems. BMC Public Health. 2019;19:512.

47. Maciejewski C, Ozierański K, Basza M, Lodziński P, Śliwczyński A, Kraj L, et al. Administrative Data in Cardiovascular Research-A Comparison of Polish National Health Fund and CRAFT Registry Data. Int J Environ Res Public Health. 2022;19:11964.

48. Maciejewski C, Ozierański K, Basza M, Barwiołek A, Ciurla M, Bożym A, et al. Practical use case of natural language processing for observational clinical research data retrieval from electronic health records: AssistMED project. Pol Arch Intern Med. 2024;134:16704.
